# Impact of *Acacia arabica* Addition on Optical, Mechanical, and Fungicidal Properties of Polymethyl Methacrylate Denture Base Material

**DOI:** 10.1055/s-0045-1810070

**Published:** 2025-08-08

**Authors:** Rania Moussa, Ahmed Yaseen Alqutaibi, Muhammad Sohail Zafar, Mai Salah El-Din

**Affiliations:** 1 Department of Substitutive Dental Sciences, College of Dentistry, Taibah University, Al Madinah, Saudi Arabia; 2 Department of Prosthodontics, College of Dentistry, Ibb University, Ibb, Yemen; 3 Department of Clinical Sciences, College of Dentistry, Ajman University, Ajman, United Arab Emirates; 4 Centre of Medical and Bio-allied Health Sciences Research, Ajman, University, Ajman, United Arab Emirates; 5 School of Dentistry, University of Jordan, Amman, Jordan; 6 Prosthodontics Department, Alexandria University Main Hospital, Alexandria, Egypt

**Keywords:** polymethyl methacrylate, denture base, gum *Acacia arabica*, antifungal agents, surface properties, *Candida albicans*

## Abstract

**Objectives:**

The aim of the study was to evaluate the optical, mechanical, and microbiological performance of heat-cured polymethyl methacrylate (PMMA) modified by adding various gum *Acacia arabica* (GA) concentrations.

**Materials and Methods:**

Specimens containing 1, 2.5, 5, 7.5, and 10% GA by weight were prepared, with control specimens containing no GA. Thirty-six rectangular samplings were assessed for color changes, flexural strength, and elastic modulus. Color measurement was conducted using a spectrophotometer. A universal testing machine measured flexural strength through a three-point bending test, and the elastic modulus was calculated. A profilometer and Vickers hardness tester evaluated the surface roughness and hardness. Antifungal activity was assessed by incubating specimens with *Candida albicans* and counting colony-forming units (CFU/mL).

**Statistical Analysis:**

Data were analyzed using the Kruskal-Wallis test for antifungal activity and optical properties and One-way ANOVA for mechanical properties at a significance level of *p* < 0.05.

**Results:**

Groups with higher GA concentrations exhibited significant color changes ( *p* < 0.001). Nonsignificant changes were reported in flexural strength, elastic modulus, or surface hardness ( *p* >0.05), although lower concentrations of GA (1 and 2.5% wt) improved surface hardness. Both 7.5% and 10% wt GA significantly increased surface roughness ( *p* < 0.001). Antifungal activity increased with GA concentration declining from 12 × 10 ^8^ CFU/mL in the control to no detectable fungal growth in the 10% wt.

**Conclusion:**

Low GA concentrations (1 and 2.5%) improved hardness with minimal surface changes, while higher levels (7.5 and 10%) significantly enhanced antifungal efficacy but compromised roughness and aesthetics. A 5% GA concentration offered a balanced compromise, necessitating further investigation.

## Introduction

Chronic atrophic candidiasis, commonly referred to as denture stomatitis (DS), is one of the most prevalent oral conditions among individuals who wear dentures. 1 The etiology of DS is multifactorial, involving both systemic and localized factors that significantly contribute to its onset and progression. Systemic disorders such as diabetes mellitus, immunosuppression, and nutritional deficiencies can impair the body's natural immune responses, increasing susceptibility to opportunistic pathogens such as *Candida albicans* . At the local level, factors including ill-fitting dentures, inadequate denture maintenance, nocturnal denture use, xerostomia, fungal infections, and a compromised oral environment create conditions that favor the proliferation of pathogenic microorganisms and the subsequent development of DS. 2

Removable dentures represent a significant risk factor in enhancing *C. albicans* colonization and the formation of persistent biofilms. 3 Imperfections of denture surface characteristics offer a conducive environment favoring the retention and proliferation of microbes. This increases the prevalence of the opportunistic pathogen *C. albicans* from 45 to 65% among healthy individuals to 60 to 100% among denture wearers. 2 Prevention against DS requires the patient's cooperation and precisely employed denture hygiene measures to minimize the adherence of microorganisms to the denture surfaces. 4 Previous studies described mechanical, chemical, or combined methods for effective denture disinfection. 5 Denture brushing is the most popular denture cleaning method 4 ; nevertheless, it increases denture base surface roughness. 5 Conversely, chemical disinfectants, although efficient in eliminating candida adherence, negatively impacted denture base mechanical properties. 6 7 The treatment of DS dictates adjusting the existing ill-fitting denture or even constructing a new denture with extra expenses, strict oral hygiene instructions, and prescribed antifungal medications. 8 Topical and systemic antifungal treatments are frequently employed 9 ; despite their availability and efficacy, DS often recurs after therapy. This may be due to the persistence of *C. albicans* biofilm on oral tissues and dentures, reduced patient compliance, failed immunity, and drug resistance from frequent and long-term antifungal use. 1

Incorporating the antifungal agents into the denture base material has been investigated as an alternative denture-disinfectant approach. This strategy aims to enhance denture hygiene and inhibit fungal adhesion and colonization, bypassing the compliance challenges associated with regular denture cleaning protocols. 10 11 The addition of metal and metal oxides into the denture base resin, 10 denture lining, 11 and tissue conditioning materials 12 enhanced the antimicrobial aspects and reduced *C. albicans* adherence and growth. Metal and metal oxide nanoparticles, such as zinc oxide (ZnO) 10 and titanium dioxide (TiO _2_ ), 11 have demonstrated potent antifungal and antibacterial activities when embedded within polymethyl methacrylate (PMMA) matrices. Their antimicrobial efficacy primarily arises from mechanisms including the generation of reactive oxygen species, disruption of microbial cell membranes, and inhibition of biofilm formation. However, despite their effectiveness, concerns remain regarding their long-term biocompatibility, potential cytotoxicity, and increased material costs.

These issues have stimulated growing interest in safer, natural alternatives that maintain antimicrobial efficacy while enhancing biocompatibility and cost-effectiveness. Natural products, such as henna, 13 thymoquinone, 14 and neem powder, 15 have been investigated. *Acacia arabica* or gum *Acacia arabica* (GA), commonly known as the Nubian acacia or babul tree, is a versatile plant species used to treat various medical conditions. 16 Its dried bark powder has a complex phytochemical profile that imposes potent antimicrobial properties. 17 18 Studies have demonstrated promising oral health applications of GA, including improved periodontal and gingival indices and enhanced outcomes in chronic periodontitis. 19 GA showed antibacterial efficacy against *Enterococcus faecalis* in root canal medications. 20 GA-containing toothpaste reduced plaque, gingivitis, and gingival bleeding compared with conventional toothpaste. 21 As a natural biopolymer filler, GA enhanced the mechanical characteristics of glass ionomer luting cement. 22 Salah El-Din et al 24 reported the positive impact of overnight immersion of differently manufactured denture base resins in GA solution (50% wt./v) on reducing *C. albicans* count.

Existing research has investigated incorporating antimicrobial agents into denture base formulations to develop potentially antimicrobial denture base resins. 13 14 15 However, concerns persist regarding the biocompatibility of such additions and their critical concentrations without adversely affecting the durability of the denture base material. The increased aging population associated with limited manual dexterity and altered oral flora accompanying systemic drugs and xerostomia highlights the importance of the denture base's built-in antimicrobial properties. This study investigated the antifungal properties of PMMA integrated with different GA concentrations and the impact on the optical and mechanical characteristics of the resin. The null hypothesis was that adding GA to the PMMA denture base would not affect optical and mechanical qualities or influence *C. albicans* count.

## Materials and Methods

This study was conducted as a laboratory-based experimental *in vitro* study designed to evaluate the effects of incorporating different concentrations of GA into PMMA denture base resin on its optical, mechanical, and antifungal properties.

The Research Ethics Committee of the Faculty of Dentistry, Alexandria University, approved this *in vitro* study prior to any research-related activities (IRB No. 00010556–IORG 0008839). The sample size was calculated using IBM SPSS Statistics version 28, assuming a 5% α error and 80% study power. The calculation was based on a one-way analysis of variance (ANOVA) to compare the means of surface hardness among six groups with different concentrations of GA added to PMMA (0% [control], 1, 2.5, 5, 7.5, and 10% by weight). The assumed group means were 0.55, 0.57, 0.51, 0.50, 0.25, and 0.16, respectively, with a pooled standard deviation of 0.23, as referenced from previous literature. 22 Based on these parameters, an effect size of 0.754 was calculated, and a total sample size of 36 specimens ( *n* = 6 per group) was determined to achieve a statistical power of 0.80 (actual power = 0.845).

### Study Design

The specimens were made of conventional heat-cured PMMA (Acrostone heat-cured denture base, © 2023 Acrostone Dental & Medical Supplies, Egypt), following the ISO standard 20795–1:2013. 23 Six groups were prepared based on the percent of GA powder included in the PMMA: 0% (control group), 1, 2.5, 5, 7.5, and 10% by weight (wt.; Table 1 ).

**Table 1:** Experimental groups description according to GA concentrations

Group	Percent of GA in the PMMA powder
Control	Unmodified acrylic resin specimens (0% GA/wt)
A	Acrylic resin specimens containing (1% GA/wt)
B	Acrylic resin specimens containing (2.5% GA/wt)
C	Acrylic resin specimens containing (5% GA/wt)
D	Acrylic resin specimens containing (7.5% GA/wt)
E	Acrylic resin specimens containing (10% GA/wt)

Abbreviations: GA, *Acacia arabica* ; PMMA, polymethyl methacrylate.

**Fig. 1:**
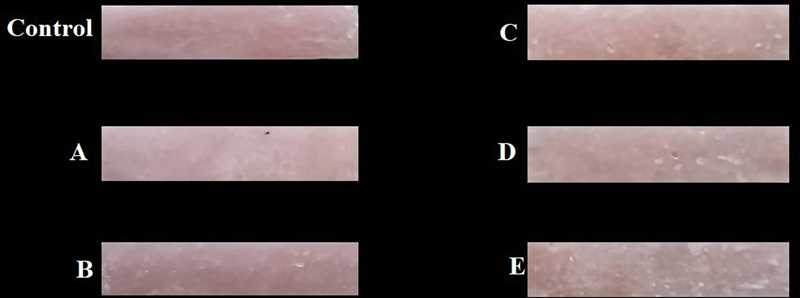
Photograph of the experimental groups: Control, ( **A** ) 1% wt GA, ( **B** ) 2.5% wt GA, ( **C** ) 5% wt GA, ( **D** ) 7.5% wt GA, and ( **E** ) 10% wt GA.

Thirty-six rectangular specimens (65 × 10 × 2.5 mm) were prepared for optical properties, flexural strength, and elastic modulus tests ( *n* = 6/group; Fig. 1 ). Following the fracture, 36 square specimens were prepared and adjusted for the surface roughness and hardness evaluations. Additionally, separate specimens were sterilized in 70% ethanol to conduct the assessment of *C. albicans* .

## Materials Preparation

Raw GA, free from additives, was collected locally. The whole gum was rinsed thoroughly with clean water to remove any impurities. The cleaned gum was spread thinly on clean, dry trays and air-dried in the shade. Once the gum was completely dry and brittle, it was ground into a fine powder and passed through a microfiltration paper. 25 An electronic balance (RADWAG Wagi Elektroniczne, Model: As 220. R2) S/N: 509946) was used to weigh the GA powder added to the PMMA polymer at ratios of 1, 2.5, 5, 7.5, and 10% wt. 13 The PMMA GA powder was vigorously stirred for 10 minutes, a procedure standardized across all batches to enhance reproducibility. 26

### Specimen Preparation

A metal template (65 × 10 × 2.5 mm) was used to create negative molds in polyvinyl siloxane (Elite HD; Zhermack SpA). Casting wax (Cavex setup modeling wax.4159002, Kandil Medical Company, Egypt) was poured into the molds to create wax patterns for tested material groups and invested in type III dental stone (SHERA extra hard dental stone: MODEL STONE GREEN, TYPE III: MJ Dental Supplies https://www.mjdentalsupplies.com.my , Selangor, Malaysia) in a metal flask. The wax was melted, and a two-part mold space was generated. Polymer and monomer were measured, mixed according to the producer's directions, and packed at the dough phase. Curing was allowed for 7 hours at 70°C and then for 1 hour at 100°C in a programmed polymerization machine (Kavo EWL 5501; Kavo Electrotechnisches Werk GmbH, Leutkirch, Germany). Then, flasks were bench-cooled before the specimens were deflasked. Silicon carbide papers of 240, 600, and 1,000 grit were sequentially used to standardize the specimen surfaces before surface testing (MicroCut PSA, Buehler, IL, United States). 26 Specimen surfaces were then polished using a felt wheel with diamond paste in moist circumstances to standardize the surface roughness (Metaserv 250 grinder-polisher; Buehler GmbH, IL, United States). A trained technician reassessed the specimen measurements to 0.01-μm accurateness by a digital caliber (NEIKO 01407A Electronic Digital Caliper, Neiko Tools USA, Greenacres, FL, United States). Finally, specimens were immersed in distilled water maintained at 37°C for 48 hours preceding to analysis. 26

### Color Measurement

A monochromator spectrophotometer supplied with computer software was used to assess the spectral radiance of the specimens of each material group (Evolution 300–UV-VIS Spectrophotometer, Thermo Fisher Scientific, Madison, WI, United States). The manufacturer's instructions were followed for device calibration. Ultraviolet and visible absorption spectrophotometry is a device that relies on measuring the reduction of electromagnetic radiation by an absorbing substance. This radiation spans a spectral range of roughly 190 to 800 nm. 27 The specimens' spectrum was then converted to the Commission Internationale de l'éclairage (CIE) color space values: L* (brightness), a* (red-green coordinate), and b* (yellow-blue coordinate) described by Alp et al. 27

The test groups' color changes were computed using the CIEDE2000 formula (∆E _00_ ).

K _L_ , K _C_ , and K _H_ equal 1. 27

where ∆L′ CIEDE2000 = lightness change,
∆C′ CIEDE2000 = chroma change,
∆H′ CIEDE2000 = hue change,
K _L_ =lightness parametric element,
K _C_ = chroma parametric element,
K _H_ = hue parametric element,
S _L_ = lightness weighting function,
S _C_ = chroma weighting function,
S _H_ = hue weighting function,
R _T_ = rotation function.

### Flexural Strength and Elastic Modulus

Flexural strength was analyzed using a three-point bending test on a universal testing machine (5 ST, Tinius Olsen, England). Specimens were positioned on three-point flexure equipment with a 50-mm support span, and a 50-kg load was directed at the midpoint at a 5 mm/min crosshead speed until the specimen fractured, at which point the fracture load was recorded. The fracture load was measured in newtons (N), then converted to megapascal (MPa) using the following formulation 26 :

FS = 3WL/2bd ^2^ ,

where FS = flexural strength (in MPa),
W = fracture load (N),
L = distance between the two supports (50 mm),
b = specimen width (10 mm),
d = specimen thickness (2.5 mm).

The flexural strength test data were used to compute the elastic modulus via the following mathematical formulation 26 :

E = FL ^3^ /4 bh ^3^ d,

where E = elastic modulus (MPa),
F = load (N) at an accessible point (p) in the straight line of the tension/deformation curve (elastic deformation),
L = distance between the two supports (50 mm),
b = sample width (10 mm),
h = sample thickness (2.5 mm),
d = deflection at point (p).

### Surface Roughness

A profilometer (MarSurf PS 10, Mahr, GmbH, Göttingen, Germany) evaluated surface roughness (Ra) in μm. The digital contact profilometer scanned the surface at three sites, one at the center and two points 3 mm from each side of the center, with a precision of 0.001 μm and a total measurement length of 0.8 mm. The three Ra (µm) evaluations were averaged for each specimen and recorded. 26

### Surface Hardness

Each specimen's Vickers hardness number (VHN) was assessed using a hardness tester (HST-HVS1000TH Micro Vickers Hardness Tester, Jinan Hensgrand Instrument Co., Ltd). The Vickers diamond indenter applied a 50-g load for 10 seconds to create a rhomboidal indentation. Three indentations were performed for each specimen, and the mean value was calculated and documented. 26

### Antifungal Assay

The test was performed at the Bioscience Microbiological Laboratory in Cairo, Egypt. *Candida albicans* (ATCC 10231) was incubated at 37°C for 48 hours in Sabouraud dextrose agar (SDA) medium (HiMedia Laboratories LLC, Pennsylvania, United States). A suspension containing 1 × 10 ^6^ colony-forming units per milliliter (CFU/mL) was prepared in Sabouraud dextrose broth (SDB) medium, employing 0.5 McFarland test standards. The optical density was adjusted to 530 nm. 28 Test specimens were sterilized by immersion in 70% ethanol for 30 minutes and then washed with sterile saline solution. 28 Subsequently, they were dipped separately in 2 mL of SDB medium contained within sterile Eppendorf tubes. Thereafter, 100 μL of the previously prepared *C. albicans* suspension was added to each tube and incubated at 25°C for 16 hours, mimicking everyday prosthesis use. 28

To regulate the sustainability of the *C. albicans* cells, culture was made from the extract of each sample into the SDA medium by extracting the resin sample that was cleaned with sterile saline, then positioned into 1-mL sterile saline and vortexed for 2 minutes to separate stuck yeast cells. A 100 μL of the formed suspension was serially diluted, then cultivated in SDA and incubated at 25°C for 48 hours. The grown colonies were counted using the Quebec colony counter, and the dilution ratio was factored in to determine the values presented as colony-forming units per milliliter (CFU/mL). 28

### Statistical Analysis

This study employed SPSS 27.0 statistical software for data analysis (IBM, New York, United States). The Shapiro–Wilk test was used to assess data normality, with *p* > 0.05 indicating a normal distribution. Descriptive statistics, presented as means and standard deviations, were calculated. One-way analysis of variance (ANOVA) test compared means of the normally distributed data, and the Kruskal–Wallis test compared the non-normally distributed data. Tukey's post hoc test compared all possible group pairs at a 95% confidence interval and significance at *p* < 0.05.

## Results

**Fig. 2:**
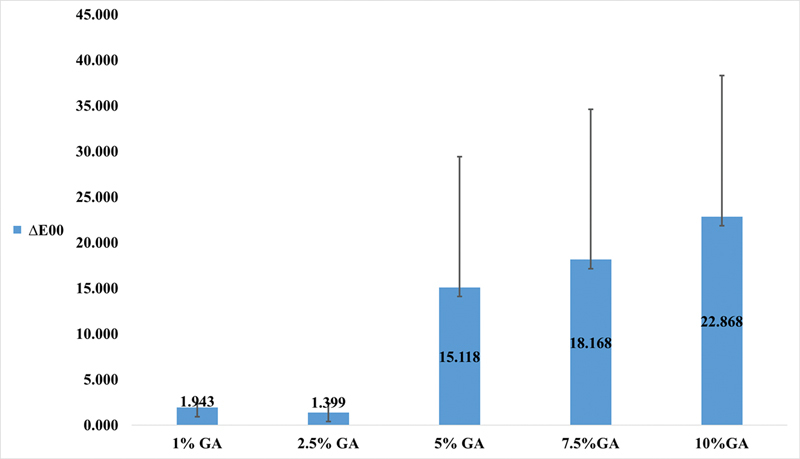
Mean and 95% confidence limits of the mean color changes CIEDE2000 (∆E _00_ ) of the test groups: 1% GA, 2.5% GA, 5% GA, 7.5% GA, and 10% GA.

Results of the color differences from the addition of different concentrations of GA to heat-cured PMMA denture base material are represented in Fig. 2 . Independent samples Kruskal–Wallis test showed that the mean ∆E _00_ of the tested groups exhibited significant differences ( *p* = 0.001). The results indicated that groups A and B demonstrated the lowest ∆E00 values (1.943 ± 0.42 and 1.39 ± 0.98, respectively) without a statistically significant difference between the two groups ( *p* = 0.67).

The highest mean ∆E00 values were observed in groups E, D, and C (22.868 ± 15.46, 18.168 ± 16.45, and 15.118 ± 14.32, respectively), with no significant differences between these three groups. In contrast, the mean ∆E00 of group A was significantly lower than those of groups C ( *p* = 0.006), D ( *p* = 0.010), and E ( *p* = 0.013). Similarly, the mean ∆E00 of group B was significantly lower than those of groups C ( *p* = 0.001), D ( *p* = 0.003), and E ( *p* = 0.003).

**Fig. 3:**
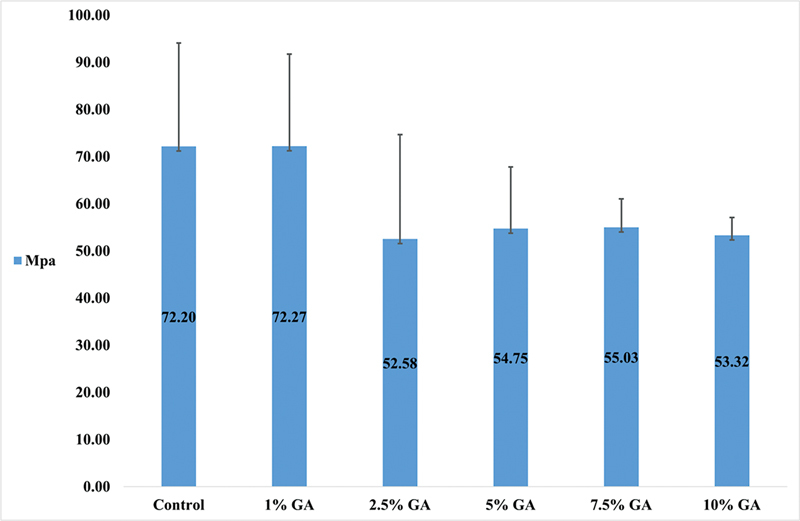
Mean and 95% confidence limits of flexural strength in megapascal (MPa) of the control and experimental groups: 1% GA, 2.5% GA, 5% GA, 7.5% GA, and 10% GA.

**Fig. 4:**
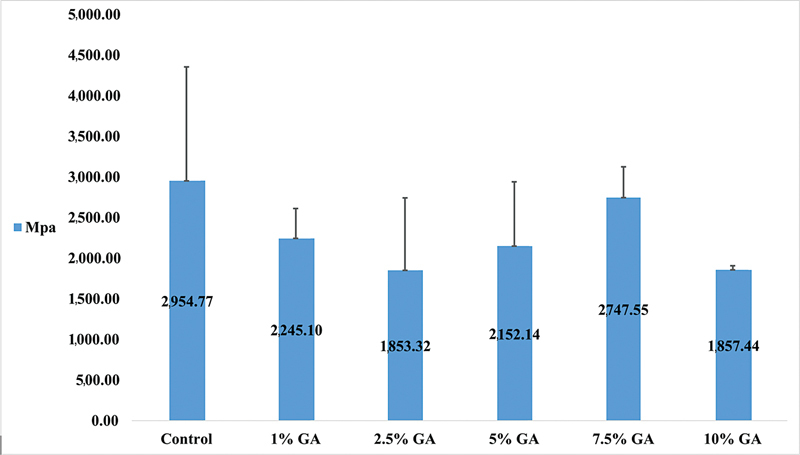
Mean and 95% confidence limits of elastic modulus in megapascal (MPa) of the control and experimental groups; 1% GA, 2.5% GA, 5% GA, 7.5% GA, and 10% GA.

Fig. 3 compares the mean flexural strength values of the study groups in MPa. Statistical analysis showed no significant differences between the trial groups ( *p* = 0.097). All pairwise comparisons were nonsignificant ( *p* >0.05). Fig. 4 compares the mean elastic modulus values of the tested groups in MPa. There were no significant differences between the experimental groups ( *p* = 0.098). All pairwise comparisons were nonsignificant ( *p* > 0.05).

The mean Ra ± standard deviations in micrometer of the experimental groups are illustrated in Table 2 . Statistical analysis displayed significant differences between the groups ( *p* < 0.001). Tukey's post hoc pairwise comparisons revealed that the groups modified with 7.5 and 10% GA had a significantly higher Ra than the control group ( *p* = 0.001 and <0.001, respectively). In contrast, no differences were reported between the control and groups A, B, and C ( *p* = 0.75, 0.824, 0.992, respectively).

**Table 2:** Mean ± standard deviation (SD) of surface roughness (Ra) in micrometer (µm) and surface hardness in VHN of each experimental group

Experimental groups	Surface roughness Ra (µm)				Surface hardness (VHN)			
	Mean ± SD	Min.	Max.	SSD	Mean ± SD	Min.	Max.	SSD
Control	0.202 ± 0.09	0.12	0.38	D, E	14.917 ± 0.32	14.33	15.33	A, B
A (1% GA)	0.129 ± 0.03	0.07	0.17	C, D, E	17.916 ± 1.15	15.33	19.33	C, D, E, control
B (2.5% GA)	0.170 ± 0.03	0.12	0.22	D, E	16.972 ± 0.64	16.00	18.33	C, D, E, control
C (5% GA)	0.218 ± 0.09	0.10	0.35	A, D, E	15.139 ± 0.94	13.67	16.33	A, B, D
D (7.5% GA)	0.320 ± 0.05	0.23	0.38	A, B, C, E, control	14.139 ± 0.80	12.67	15.00	A, B, C
E (10% GA)	0.417 ± 0.04	0.33	0.49	A, B, C, D, control	14.278 ± 0.72	13.00	15.33	A, B
*p* -value	< 0.001				< 0.001			

Abbreviations: GA, *Acacia arabica* ; Max, maximum; Min, minimum; SSD, statically significant difference between groups at *p* < 0.05; Std. dev., standard deviation; VHN, Vickers hardness number.

Table 2 also presents the statistical analysis of the VHN. ANOVA revealed a significant difference between the tested groups ( *p* < 0.001). The mean VHN values were significantly higher in groups A and B than in the control group ( *p* < 0.001). In contrast, no significant differences were observed when comparing groups C, D, and E to the control group ( *p* = 0.984, 0.181, and 0.382, respectively).

**Fig. 5:**
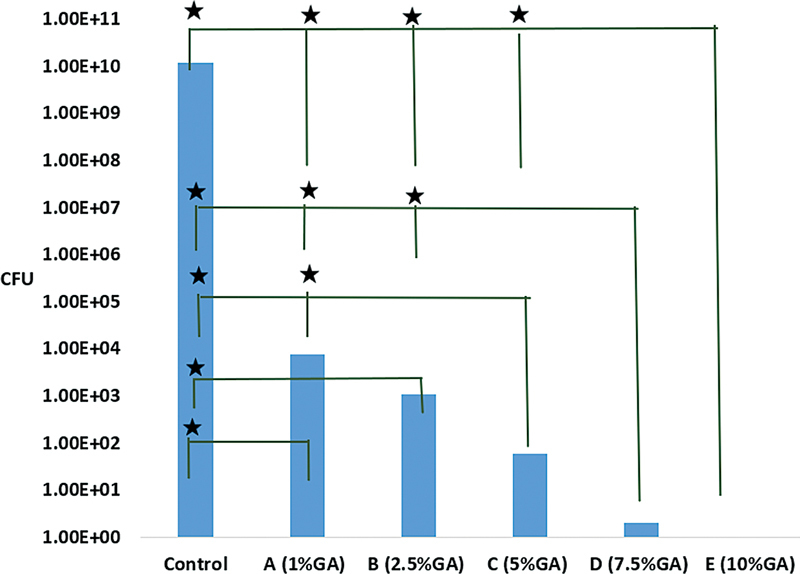
Colony forming units (CFU)/mL of *Candida albicans* on SDA medium: ( **A** ) 1% GA, ( **B** ) 2.5% GA, ( **C** ) 5% GA, ( **D** ) 7.5% GA, and ( **E** ) 10% GA. *Significant differences between groups, *p* < 0.05.

**Fig. 6:**
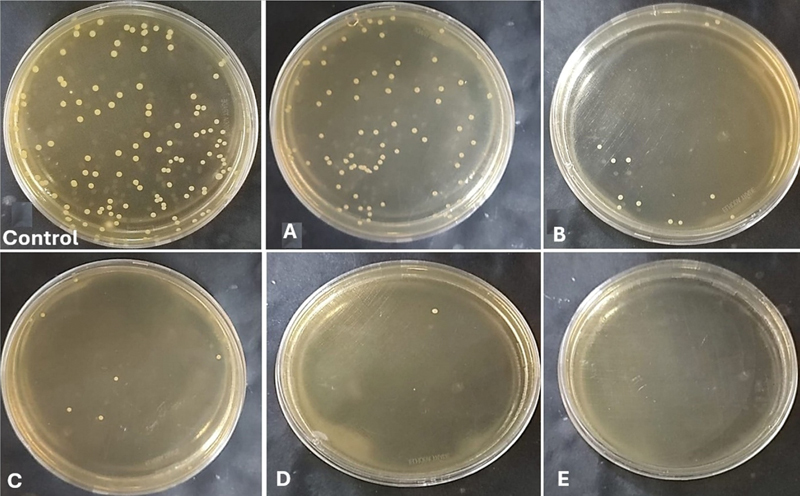
*Candida albicans* colonies on Sabouraud dextrose agar plates: control, ( **A** ) 1% wt GA, ( **B** ) 2.5% wt GA, ( **C** ) 5% wt GA, ( **D** ) 7.5% wt GA, and ( **E** ) 10% wt GA.

Fig. 5 compares *C. albicans* CFU per milliliter of the tested material groups. PMMA denture base specimens exhibited a statistically significant decrease in *C. albicans* cell count with increasing GA concentration ( *p* < 0.001). Notably, the *Candida* count decreased substantially from 121 × 10 ^8^ CFU/mL in the control group to 78 × 10 ^2^ , 11 × 10 ^2^ , 60, 1, and 0 CFU/mL in groups A, B, C, D, and E, respectively ( Fig. 6 ).

## Discussion

The existing study investigated the optical, mechanical, and antifungal properties of an innovative PMMA denture base material modified with varying GA concentrations. GA significantly affected the optical and antifungal properties of the resin. Only specific mechanical parameters (hardness and roughness) differed at certain GA levels, while flexural strength and modulus of elasticity remained unaffected. Consequently, the null hypotheses regarding optical properties and antifungal efficiency were rejected, while that of the mechanical properties was partially accepted.

The current study utilized an objective approach, using noncontact spectrophotometry, to assess color differences in the modified PMMA resin. The Commission Internationale de l'éclairage L*, a*, b* system, which includes a full spectrum of visible colors, was applied as an appropriate instrument for evaluating the color changes. 29 Perceptibility and acceptability thresholds reported by Ren et al were applied, 29 with 1.72 CIEDE2000 units representing the perceptibility threshold and 4.08 CIEDE2000 units denoting acceptability for the color changes for 50% of the viewers. 27 The findings of the current study indicated that adding GA at 5% wt or higher led to substantial color changes in PMMA denture base material exceeding the acceptable limit. Adding the GA powder increased the b* values in the mentioned groups, indicating a shift of the material color toward the yellow spectrum. This suggests that the aesthetics of PMMA-based materials may be compromised when modified with elevated GA concentrations, potentially impacting patients' appearance and satisfaction with dental prostheses. The color alterations observed at GA concentrations of 5% and above may restrict the application of altered resin in highly aesthetic zones. Nevertheless, such formulations remain suitable for use in posterior dentures or palatal extensions, where aesthetic demands are less critical and the enhanced antifungal properties induced by GA incorporation are particularly advantageous, such as those with a history of recurrent DS. Notably, the color difference observed in the 5% GA group falls within a range that may be amenable to correction using established extrinsic staining and characterization techniques. 30 31 Nevertheless, although extrinsic color adjustment remains a viable option, it necessitates additional clinical procedures and may not fully restore the original aesthetic appearance. This underscores the importance of future research to systematically evaluate the feasibility and effectiveness of such interventions in clinical settings. High standard deviations observed in ∆E00 measurements are acknowledged and may be attributed to the natural variability of GA powder and its dispersion within the PMMA matrix. Thus, although the GA powder was manually mixed for a fixed time using a mortar and pestle to achieve uniform distribution, we acknowledge that this method may not ensure homogeneity, particularly at higher GA concentrations. Future studies are recommended to incorporate quantitative microstructural evaluation techniques to assess filler dispersion and its relationship with mechanical and surface properties, thereby reducing optical variability.

Masticatory forces are a recurring phenomenon that can lead to substantial cracking of denture base material. Furthermore, as the denture rests on uneven alveolar ridges resulting from continuous bone resorption, the base could weaken and eventually fracture. Thus, fracture strength and elastic modulus are crucial when evaluating the clinical durability of a denture base. 32 Adding GA to PMMA did not affect the flexural strength and elastic modulus of the PMMA, with reported average values that complied with the thresholds recommended by ISO Standard 20795–1:2013 of 65 and 2,000 MPa. 24 Factors such as shape and filler size, aspect ratio, particle alignment, agglomeration, interphase, and particle loading are influential. 26 33 The findings of this investigation differed from those of previous reports by Alsadon et al 25 and Khan et al. 32 They reported that the flexural strength and modulus of PMMA were inversely proportional to the GA content. The variation may be due to the changed proportions and sources of GA. Furthermore, they soaked the GA powder in 3-methylacryloxyproyltrimethoxysilane using a silane coupling agent before adding it to the PMMA powder, which may have affected the spread within the PMMA powder or the interaction with the PMMA lattice. 32

The inherent surface roughness of PMMA denture base material makes it susceptible to *C. albicans* settlement and biofilm establishment, emphasizing the need for a smoother surface to minimize microbial attachment. 2 It has been reported that Ra of 0.2 µm is the clinically accepted threshold, beyond which the denture offers a considerable surface area for microbial colonization. 26 Adding GA in minimal concentrations to the denture resin did not roughen its surface; conversely, GA concentrations exceeding 5% substantially increased surface irregularities. This could be attributed to the poor chemical bond of GA to PMMA resin; thus, as GA clusters on the surface, it can be readily dislodged during finishing and polishing, resulting in surface pores. 32 These findings align with those of Khan et al. 32 Their study results showed that the surface topography of the PMMA modified by low GA concentrations (≤5%) met the minimum clinically acceptable Ra threshold.

Hardness renders the material resistant to abrasion and signifies easy finishing and endurance to scratches during cleaning. The microhardness data indicate that incorporating GA powder at low concentrations improves the hardness of PMMA, whereas at elevated concentrations, the surface hardness remains unaffected. This effect can be attributed to the more uniform dispersion of GA particles within the PMMA matrix at low concentrations, allowing GA to act effectively as a micro-filler. Such uniform distribution likely enhances matrix densification and reduces free volume, improving the resistance to surface deformation. 32 Conversely, at higher concentrations (≥5%), probably GA particle agglomeration and poorer filler–matrix interfacial bonding may lead to stress concentrations and weak spots that offset any potential reinforcing benefits. This hypothesis is indirectly supported by the significant increase in surface roughness observed in the 7.5 and 10% groups, which may result from loosely bound or dislodged GA clusters during the finishing and polishing processes. Without direct microstructural evaluation (e.g., scanning electron microscopy [SEM] or energy-dispersive X-ray spectroscopy [EDS]), this interpretation remains speculative but consistent with studies by Alsadon et al 25 and Khan et al. 32 They reported negligible differences in microhardness between the control group and samples containing 5 and 10% wt GA. However, they observed a substantial decrease in microhardness for PMMA modified with 20% GA, recommending that the impact of GA powder be investigated at lower concentrations, as done in the current research.

Modifications of conventional PMMA denture base materials are commonly reported in the literature. 34 35 Modification of the PMMA denture base material with different GA concentrations revealed a considerable decrease in the CFU/mL of *C. albicans* . The antifungal efficacy increased in proportion to the GA concentration. Even the 1% wt addition of GA substantially reduced *C. albicans* colony formation, and completely eradicated fungal colonies was observed at 10% wt. Previous research has shown that GA is rich in various phytochemicals, including tannins, glycosides, alkaloids, flavonoids, saponins, phenols, and terpenoids. 18 36 These diverse metabolites have demonstrated potent inhibitory effects against a wide range of microbial pathogens. 18 Tannins in GA can disrupt microbial cell membranes. At the same time, saponins and flavonoids can interfere with essential cellular processes, leading to growth inhibition and even cell death of bacteria, fungi, and viruses. Similarly, alkaloids and phenolic compounds possess potent antioxidant and antimicrobial activities, making this natural gum a promising source of bioactive compounds for the development of novel antimicrobial agents. 36 Owing to the novelty of the material used in this study, it was challenging to find comparable research to validate the results. Nevertheless, the current results align with those of Nawasrah et al, 13 Al-Thobity et al, 14 and Hamid et al, 15 who demonstrated that plant extracts can inhibit *C. albicans* adhesion at various concentrations.

When evaluating the overall performance of GA-modified PMMA, it becomes apparent that no single concentration provides a definite improvement across all tested parameters. At 1 and 2.5% GA, hardness increased significantly with minimal aesthetic and surface alterations; however, antifungal efficacy remained limited. At 5% GA, a balance was achieved and antifungal activity was markedly improved, with surface roughness approaching the clinical threshold of 0.2 µm, and without statistically significant deterioration of mechanical properties. At 7.5 and 10% GA, antifungal efficacy peaked, but surface quality and aesthetics were compromised. These results suggest that 5% GA may be a clinically acceptable compromise, rather than an absolute optimum, depending on the specific clinical priority (e.g., antifungal protection vs. mechanical integrity).

The findings of the current study require cautious interpretation. *In vitro* experiments often use standardized and basic settings that may not fully mimic the complex oral conditions such as testing antifungal activity of one *Candida* strain and applying a three-point flexural strength test, which applies unidirectional force, while intraorally, multidirectional and rotational forces are experienced. 25 Although GA powder was air-dried, ground, and filtered using microfiltration paper to achieve a fine consistency, the precise particle size and its distribution were not measured, and potential clustering, especially at higher concentrations, cannot be ruled out. The current study demonstrated concentration-dependent trends in hardness and surface roughness, and it did not employ microstructural characterization techniques, such as SEM or EDS, to confirm the distribution of GA within the PMMA matrix.

Accordingly, future studies are warranted to incorporate SEM imaging and spectroscopic analysis of fractured or polished cross-sections to visualize filler dispersion and investigate interfacial interactions between GA and the PMMA resin. Such analyses would provide valuable insights into the structure–property relationships underlying the performance of GA-modified denture base materials. Additionally, aging investigations are recommended to assess the endurance and strength of GA-modified PMMA under prolonged cycling conditions. Studies on nano- and micron-sized GA powder should be considered to examine the strengthening of the denture base polymer while producing a smooth surface and to determine optimal GA concentrations tailored to specific clinical requirements.

This study focused solely on GA as a standalone natural additive. Future investigations should include comparative studies assessing GA against both inorganic agents (e.g., ZnO, TiO _2_ nanoparticles) and other plant-based antifungals. Such studies would offer a comprehensive understanding of the relative efficacy, cytocompatibility, and cost-effectiveness of natural versus nanoparticle-based denture base modifications.

## Conclusion

Within the limitations of this *in vitro* study, the incorporation of GA into the PMMA denture base resin demonstrated variable effects depending on the concentration. At lower concentrations (1 and 2.5%), GA enhanced surface hardness and maintained favorable optical and surface characteristics but yielded modest antifungal activity. In contrast, higher concentrations (7.5 and 10%) maximized antifungal efficacy yet compromised surface roughness and aesthetics. A concentration of 5% GA offered a pragmatic compromise, showing improved antifungal action with tolerable surface changes and no significant deterioration in flexural strength or modulus. Therefore, rather than identifying a singular “optimal” concentration, the results support a multifaceted consideration of clinical priorities, including mechanical durability, surface quality, aesthetics, and microbial resistance. Further research integrating microstructural characterization, long-term aging, and clinical simulation is needed to confirm the optimal performance range for GA-modified PMMA resins in practical settings.

## References

[1] Abuhajar E Ali K Zulfiqar G Management of chronic atrophic candidiasis (denture stomatitis): a narrative review Int J Environ Res Public Health 2023 20 04 3029 36833718 10.3390/ijerph20043029 PMC9967389

[2] Perić M Miličić B Kuzmanović Pfićer J Živković R Arsić Arsenijević V a systematic review of denture stomatitis: predisposing factors, clinical features, etiology, and global *Candida* spp. distribution J Fungi (Basel) 2024 10 05 328 38786683 10.3390/jof10050328 PMC11122031

[3] Sakr H M AbdulSalam M R Fayad M I Moussa R Alzahrani A AH Microbial adhesion to different thermoplastic denture base materials in Kennedy class I partially edentulous patients Cureus 2024 16 05 e60421 38756717 10.7759/cureus.60421 PMC11097705

[4] Moussa R Alruhailie L GA Saleh S AM Assessment of denture hygiene knowledge and attitude in Al Madinah AlMunawwarah J Int Dent Med Res 2022 15 02 814 819

[5] Fouda S M Gad M M Ellakany P Influence of denture brushing on the surface properties and color stability of CAD-CAM, thermoformed, and conventionally fabricated denture base resins J Prosthodont 2025 34 01 91 100 37953735 10.1111/jopr.13801

[6] Moussa R Ellakany P Fouda S M El-Din M S Comparative evaluation of the effects of laser and chemical denture disinfectants on the surface characteristics of CAD-CAM and conventional denture resins: An in vitro experimental study J Prosthodont 2024 1 9 10.1111/jopr.13952 PMC13456428 39300670

[7] Alkaltham N S Aldhafiri R A Al-Thobity A M Effect of denture disinfectants on the mechanical performance of 3D-printed denture base materials Polymers (Basel) 2023 15 05 1175 36904416 10.3390/polym15051175 PMC10007094

[8] Mylonas P Milward P McAndrew R Denture cleanliness and hygiene: an overview Br Dent J 2022 233 01 20 26 35804119 10.1038/s41415-022-4397-1 PMC9270218

[9] Lu S Y Oral candidosis: pathophysiology and best practice for diagnosis, classification, and successful management J Fungi (Basel) 2021 7 07 555 34356934 10.3390/jof7070555 PMC8306613

[10] Kamonkhantikul K Arksornnukit M Takahashi H Antifungal, optical, and mechanical properties of polymethylmethacrylate material incorporated with silanized zinc oxide nanoparticles Int J Nanomedicine 2017 12 2353 2360 28392692 10.2147/IJN.S132116 PMC5376186

[11] Ahmed A Q Al-Hmedat S JA Hanweet D M Haider J Assessing the antifungal activity of a soft denture liner loaded with titanium oxide nanoparticles (TiO _2_ NPs) Dent J 2023 11 04 90 10.3390/dj11040090 PMC10137426 37185468

[12] Iqbal Z Zafar M S Role of antifungal medicaments added to tissue conditioners: a systematic review J Prosthodont Res 2016 60 04 231 239 27085676 10.1016/j.jpor.2016.03.006

[13] Nawasrah A AlNimr A Ali A A Antifungal effect of henna against *Candida albicans* adhered to acrylic resin as a possible method for prevention of denture stomatitis Int J Environ Res Public Health 2016 13 05 520 27223294 10.3390/ijerph13050520 PMC4881145

[14] Al-Thobity A M Al-Khalifa K S Gad M M Al-Hariri M Ali A A Alnassar T *In vitro* evaluation of the inhibitory activity of thymoquinone in combatting *Candida albicans* in denture stomatitis prevention Int J Environ Res Public Health 2017 14 07 743 28698449 10.3390/ijerph14070743 PMC5551181

[15] Hamid S K Al-Dubayan A H Al-Awami H Khan S Q Gad M M *In vitro* assessment of the antifungal effects of neem powder added to polymethyl methacrylate denture base material J Clin Exp Dent 2019 11 02 e170 e178 30805122 10.4317/jced.55458 PMC6383901

[16] Kumar A Kumar S Singh M K Tiwari S K A comprehensive review on the chemical composition and pharmacological activities of *Acacia arabica* Intell Pharm 2024 2 05 729 736

[17] Ramalingam K Amaechi B T Antimicrobial effect of herbal extract of *Acacia arabica* with triphala on the biofilm forming cariogenic microorganisms J Ayurveda Integr Med 2020 11 03 322 328 30389224 10.1016/j.jaim.2018.01.005 PMC7527819

[18] Baien S H Seele J Henneck T Antimicrobial and immunomodulatory effect of gum Arabic on human and bovine granulocytes against *Staphylococcus aureus* and *Escherichia coli* Front Immunol 2020 10 3119 32082302 10.3389/fimmu.2019.03119 PMC7005937

[19] Singhal R Agarwal V Rastogi P Khanna R Tripathi S Efficacy of *Acacia arabica* gum as an adjunct to scaling and root planing in the treatment of chronic periodontitis: a randomized controlled clinical trial Saudi Dent J 2018 30 01 53 62 30166872 10.1016/j.sdentj.2017.10.006 PMC6112319

[20] Mohamed A A Fayyad D M El-Telbany M Mohamed D AA Antibacterial biofilm efficacy of calcium hydroxide loaded on gum Arabic nanocarrier: an in-vitro study BMC Oral Health 2024 24 01 215 38341565 10.1186/s12903-024-03941-3 PMC10859034

[21] Tangade P S Mathur A Tirth A Kabasi S Anti-gingivitis effects of *Acacia arabica* -containing toothpaste Chin J Dent Res 2012 15 01 49 53 22866283

[22] Khan A A Bari A Abdullah Al-Kheraif A Oxidized natural biopolymer for enhanced surface, physical and mechanical properties of glass ionomer luting cement Polymers (Basel) 2023 15 12 2679 37376329 10.3390/polym15122679 PMC10301862

[23] International Organization for Standardization. ISO 20795–1. Dentistry-Base Polymers-Part 1: Denture Base Polymers Geneva, Switzerland International Organization for Standardization 2013

[24] Salah El-Din M Shehat M G Kamal S M Potential antifungal effect of *Acacia arabica* extract versus sterile water regarding surface topography of denture base materials: an *in-vitro* study Alex Dent J 2024 50 02 116 122

[25] Alsadon O Alkhureif A A Khan A A Effect of gum Arabic powder on the mechanical properties of denture base acrylic Pak J Med Sci 2023 39 01 223 226 36694769 10.12669/pjms.39.1.6937 PMC9842989

[26] Gad M M Al-Thobity A M Fouda S M Näpänkangas R Raustia A Flexural and surface properties of PMMA denture base material modified with thymoquinone as an antifungal agent J Prosthodont 2020 29 03 243 250 30178899 10.1111/jopr.12967

[27] Alp G Johnston W M Yilmaz B Optical properties and surface roughness of prepolymerized poly(methyl methacrylate) denture base materials J Prosthet Dent 2019 121 02 347 352 30143239 10.1016/j.prosdent.2018.03.001

[28] Türkcan İ Nalbant A D Bat E Akca G Examination of 2-methacryloyloxyethyl phosphorylcholine polymer coated acrylic resin denture base material: surface characteristics and *Candida albicans* adhesion J Mater Sci Mater Med 2018 29 07 107 29971499 10.1007/s10856-018-6116-7

[29] Ren J Lin H Huang Q Zheng G Determining color difference thresholds in denture base acrylic resin J Prosthet Dent 2015 114 05 702 708 26277020 10.1016/j.prosdent.2015.06.009

[30] Goiato M C Zuccolotti B CR dos Santos D M Sinhoreti M AC Moreno A Effect of intrinsic nanoparticle pigmentation on the color stability of denture base acrylic resins J Prosthet Dent 2013 110 02 101 106 10.1016/s0022-3913(13)60387-x 25564691

[31] Park B W Kim N J Lee J Lee H H Technique for fabricating individualized dentures with a gingiva-shade composite resin J Prosthet Dent 2016 115 05 547 550 26794697 10.1016/j.prosdent.2015.11.010

[32] Khan A A Alkhureif A A Awaiyer M S Bautista L SJ Surface, mechanical and chemical properties of modified denture resin using natural biopolymer Pak J Med Sci 2023 39 06 1631 1636 37936770 10.12669/pjms.39.6.7837 PMC10626063

[33] Vattathurvalappil S H Haq M Kundurthi S Hybrid nanocomposites: an efficient representative volume element formulation with interface properties Polym Polymer Compos 2022 30 1 13

[34] Zafar M S Prosthodontic applications of polymethyl methacrylate (PMMA): an update Polymers (Basel) 2020 12 10 2299 33049984 10.3390/polym12102299 PMC7599472

[35] Hassan M Asghar M Din S U Zafar M S Thermoset polymethacrylate-based materials for dental applications London Elsevier 2019 273 308

[36] Munir H Bilal M Khan M I Iqbal H MN Gums-based bionanostructures for medical applications Hoboken, NJ Wiley 2021

